# *Bacillus atrophaeus* WZYH01 and *Planococcus soli* WZYH02 Improve Salt Tolerance of Maize (*Zea mays* L.) in Saline Soil

**DOI:** 10.3389/fpls.2022.891372

**Published:** 2022-05-06

**Authors:** Yaling Hou, Wenzhi Zeng, Chang Ao, Ying Luo, Zhao Wang, Menglu Hou, Jiesheng Huang

**Affiliations:** ^1^State Key Laboratory of Water Resources and Hydropower Engineering Science, Wuhan University, Wuhan, China; ^2^State Key Laboratory of Hybrid Rice, Institute for Advanced Studies, Wuhan University, Wuhan, China

**Keywords:** maize, PGPR, soil salinity, plant growth promotion, antioxidant

## Abstract

With the increasing shortage of land resources and people’s attention to the ecological environment, the application of microbial fertilizer with natural soil microorganisms as the main component has attracted increasing attention in saline agriculture. In this study, two salt-tolerant strains, YL07 (*Bacillus atrophaeus*) and YL10 (*Planococcus soli*), were isolated from maize (*Zea mays* L.) rhizosphere soil with a saturated conductivity (EC_e_) of 6.13 dS/m and pH of 8.32 (Xinjiang, China). The effects of *B. atrophaeus* WZYH01 (YL07) and *Planococcus soli* WZYH02 (YL10) on the growth and development of maize (*Zea mays* L.) under salt stress (EC_e_ = 5.9 dS/m) were further studied. The results showed that compared with uninoculation, inoculation with *B. atrophaeus* WZYH01 and *Planococcus soli* WZYH02 significantly improved maize growth performance, biomass yield, and antioxidant levels under salt stress, and the effect of *Planococcus soli* WZYH02 was more prominent than the effect of *B. atrophaeus* WZYH01. Moreover, inoculation with *B. atrophaeus* WZYH01 and *Planococcus soli* WZYH02 protected maize from salt stress by regulating plant hormone [IAA and abscisic acid (ABA)] levels and increasing nutrient acquisition. In addition, the tested strains were most efficient for maize growth and health, increasing the content of K^+^ accompanied by an effective decrease in Na^+^ in maize tissues. The transcription levels of salt tolerance genes (*ZMNHX1*, *ZMNHX2*, *ZMHKT*, *ZMWRKY58*, and *ZMDREB2A*) in inoculated maize were also dramatically higher than the transcription levels of the specified salt tolerance genes in uninoculated maize. In conclusion, *B. atrophaeus* WZYH01 and *Planococcus soli* WZYH02 can alleviate the harmful effects of salt stress on crop growth, thereby promoting sustainable agricultural development.

## Introduction

Currently, approximately 1,125 million hectares of land worldwide are affected by salinization (Wicke et al., 2011; Dong et al., 2022). In China, approximately 3.67 million hectares of soil, which represents 4.88% of the total available land across the country, is threatened by salt (Zeng et al., 2016; Liu et al., 2020). To increase crop yield in salinized land, a large amount of chemical fertilizer is applied to the soil (Wang L. L. et al., 2018), but the increase in chemical input is not directly proportional to the increase in crop yield (Hawkesford, 2014). Meanwhile, this behavior has also brought serious soil ecological and environmental problems such as secondary salinization, soil consolidation, soil acidification, and microecological imbalance (Hawkesford, 2014; Ma et al., 2021). In addition, the Ministry of Agriculture and Rural Affairs of the People’s Republic of China (2015)^1^ stated that the use of chemical fertilizer should be greatly reduced and that resource-saving and environmentally friendly modern agricultural development roads should be actively explored. Therefore, it is imperative to reduce the use of chemical fertilizer in agricultural production in saline land.

To overcome this issue, a new biocontrol approach has been developed to protect plants from salt stress in soil by utilizing beneficial microorganisms to achieve eco-friendly sustainable agriculture (Etesami and Maheshwari, 2018; Moreira et al., 2019). Plant growth-promoting rhizobacteria (PGPR) are an important microbial community that has a beneficial impact on plant growth and development. PGPR extensively colonizes plant roots, increases their growth, and reduces plant diseases (Shameer and Prasad, 2018). When combined with roots and other tissues, PGPR improves the nutritional supply of crop plants through several mechanisms. PGPR has direct effects, including nitrogen fixation, phosphorus solubilization, production of NH_3_, indole acetic acid (IAA), and siderophores, and indirect effects, including antioxidant defense, volatile organic compounds (VOCs), exopolysaccharides (EPS), and osmotic balance mechanisms for improving plant growth and enhancing tolerance against salt stress (Moreira et al., 2016; Abbas et al., 2019; Kumar et al., 2019). Therefore, for decades, people have been studying how soil microorganisms play a role in plant growth to provide an alternative way to reduce the amount of fertilizer.

The application of PGPR to alleviate salinity-induced plant stress has become a promising approach. PGPR-mediated tolerance has been demonstrated in different plant–microbe interactions (Goswami et al., 2014; Li et al., 2020; Tirry et al., 2021). However, the efficiency of PGPR is affected by environmental factors such as climate, weather conditions, soil characteristics (e.g., texture, pH, temperature, and water content), and interaction with soil indigenous microbial flora (Siddikee et al., 2010; Qiu et al., 2019; Khalilpour et al., 2021). van Elsas et al. (1986) also reported that the numbers of *Bacillus subtilis* decreased rapidly in loamy sand and silt loam, while *Pseudomonas fluorescens* survived better in silty sand than in loamy loam. In addition, Habibi et al. (2019) found that PGPR isolated from Bala Doshi rice cultivars could promote Bala Doshi rice plant growth more than other rice cultivars, which might be due to the host specificity of Bala Doshi rice cultivars to the obtained strains. Simultaneously, recent studies have increasingly emphasized the benefits of using local microorganisms to enhance plant resistance to biological and abiotic stresses (Marulanda et al., 2009; Banerjee et al., 2017), suggesting that the activities of strains already adapted to the plant environment may increase the chances of inoculum survival and confer a positive effect on plant development under stress (Qiu et al., 2019). Therefore, we suggest screening of PGPR suitable for local soil and climate conditions.

Xinjiang is one of the most important agricultural production areas in the arid and semiarid regions of China (Wang et al., 2012), but 31.1% of the existing arable land in Xinjiang has suffered soil salinization (Liang et al., 2021; Liu Y. et al., 2021). The Statistical Yearbook of Xinjiang (2015) has shown that the annual yields of these three crops (grain, vegetables, and fruits) are approximately 1.50 × 10^7^, 1.93 × 10^7^, and 0.96 × 10^7^ tons, respectively (Liu W. G. et al., 2021). At present, many studies on screening indigenous PGPR have also been carried out in these crops. Han et al. (2021) indicated that *Pseudomonas* SCPG-7 inoculated from pepper (*Capsicum annuum* L.) seeds had the ability to promote plant growth by secreting organic acids, alkaline phosphatase, siderophore, and IAA under salt stress in Shihezi, Xinjiang Province. The bacterial strain *Klebsiella oxytoca* Rs-5 was screened from the salinized soil of cotton in Xinjiang Province and could relieve salt stress and promote cotton seedling growth (Yue et al., 2007). *Pseudomonas putida* Rs-198 isolated from a cotton alkaline soil in Xinjiang mitigated osmotic stress in cotton seedlings, which led to an improved germination rate, healthy stands, and growth parameters (Yao et al., 2010). Liu et al. (2011) also reported that *Bacillus subtilis* SL-13 obtained from tomato field soil in Xinjiang Province was proven to promote sprouting and seedling growth in tomatoes. However, in the saline soils of Xinjiang, relatively few studies have been carried out on the screening of maize rhizosphere growth-promoting bacteria, which requires attention from the scientific community.

Compared with PGPR isolated from other soils, PGPR isolated from plants grown under chronically stressful salinity conditions have a stronger ability to survive due to their adaptation to the local environment (Kumar et al., 2017). However, knowledge of the potential of native PGPR isolates and their effects in maize plant growth and physiological characteristics under salt stress is still gravely limited. Therefore, the purpose of this study was to screen indigenous stress-tolerant PGPR with growth-promoting traits and evaluate the effects of PGPR isolates on the growth, physiology, and expression levels of stress-tolerant genes in maize seedlings under salt stress.

## Materials and Methods

### Plant Material, Growing Conditions, and Treatments

Two strains (YL07 and YL10) were screened from maize rhizosphere soil in Yanqi, Xinjiang province, China (41°91’ N, 86°49’ E, elevation 1,061 m). The screening method is described in detail in the Supplementary Material. Maize seeds were treated with 75% ethanol for 30 s, sterilized with 10% sodium hypochlorite solution for 15 min, and then washed with sterile water 5–6 times. Sterilized seeds were soaked in sterile water for 12 h and then dipped in strain inoculum for 2 h. All the above experiments were carried out in a sterile environment. Thereafter, the treated maize seeds were cultivated in the seedling tray for 7 days, and then, maize seedlings with consistent growth were selected and transplanted into pots containing 1.2 kg sterilized soil (120°C, 30 min, 100 kPa, and sterilized twice) in each pot. Three maize plants were maintained in each pot. The three treatments were as follows: uninoculated strain exposed to 5.9 dS/m salt stress (CK); YL07 strain exposed to 5.9 dS/m salt (YL07); and YL10 strain exposed to 5.9 dS/m salt stress (YL10). Each treatment had five repetitions. The maize was harvested after 31 days of growth.

### Growth, Biomass Yield, Physiological Indicators, and Plant Hormones

After 31 days of cultivation, the plants were collected, and the roots were rinsed with sterile water. Plant height was measured and recorded. To obtain plant dry biomass, the plants were dried at 105°C for 30 min and kept at 75 ± 2°C for 24 h to achieve a constant dry weight (DW). The leaf superoxide dismutase (SOD), catalase (CAT), peroxidase (POD), ascorbate peroxidase (APX), glutathione reductase (GR) and reduced glutathione (GSH), soluble sugar, proline, IAA, and abscisic acid (ABA) contents were measured by Qingdao Sci-tech Innovation Quality Testing Co., Ltd^2^.

### Determination of N, K^+^, Na^+^, and K^+^/Na^+^

After 31 days of cultivation, the maize roots were washed and wiped dry to determine the content of nutrients and ions. Briefly, the maize leaves and roots were ground into fine powder, and then, 0.2 g samples were weighed into digestive tubes with 1 ml of distilled water. Then, 5 ml of H_2_SO_4_ was added to the mixture, and 2 ml of hydrogen peroxide was added twice. After the fierce reaction, the mixtures were put on the digestion furnace for digestion, and heating was stopped when the solution turned brown. After cooling slightly, 10 drops of H_2_O_2_ were added, heating was continued until the solution was colorless or clear, and heating continued for 5 min to remove excess H_2_O_2_. Nutrient nitrogen (N) was determined by the Kjeldahl method as indicated previously (Bremner, 2009). K^+^ and Na^+^ were determined by flame photometry according to Wolf (2008). The K^+^/Na^+^ ratio was calculated in line with the K^+^ and Na^+^ concentrations.

### Transcription Analysis

The expression levels of related genes in maize seedlings inoculated with YL07, YL10, and the control treatment under salt stress were determined by quantitative real-time polymerase chain reaction (qRT-PCR). Total RNA was extracted from each maize seedling using TRIzol reagent following the manufacturer’s instructions. The transcription levels of the ion balance-related genes *ZmNHX1* and *ZmNHX2* and *ZmHKT*, key transcription factor genes linked to the plant response to abiotic stress *ZmDREB2A* and *ZmWRKY58*, and key genes associated with ABA synthesis *ZmNCED* were measured. The sequences of primers used in qRT-PCR to study the relative expression of genes in maize lines are presented in the Supplementary Table 1. All PCR experiments were performed with SYBR Green Master Mix on a CFX Connect Real-Time PCR Detection System (1855201) with 40 cycles, and an annealing temperature of 55°C was used (in a final volume of 20 μl). The constitutively expressed β-actin gene was used as an internal control. The relative expression levels of the target genes were calculated using the 2^–ΔΔCt^ method. Five mRNA samples from five independent leaf samples (biological replicates) were analyzed.

### Data and Statistical Analysis

All the data were statistically analyzed using RStudio (version 4.0.3). The significance of the differences between the control and treated groups was analyzed using one-way ANOVA followed by LSD *post hoc* comparison tests. Different letters indicate significant differences at the *P* < 0.05 level. Means and standard errors for all parameters were calculated from at least three replicates. Hierarchical clustering was performed using the “pheatmap” package based on Euclidean distance. Principal component analysis (PCA) was conducted using the “FactoMineR” package. All results were visualized using the “ggplot2” package in RStudio.

## Results

### Growth and Biomass Yield

**FIGURE 1:**
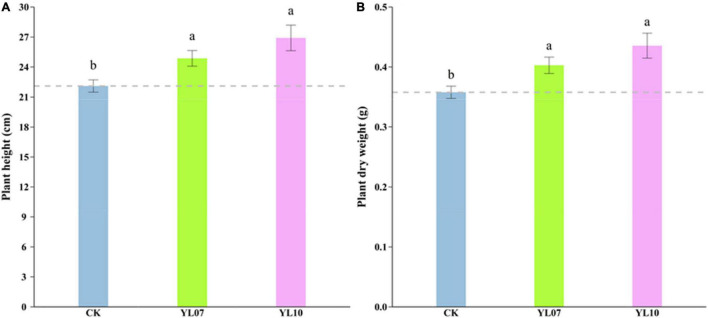
Effect of strains YL07 and YL10 on the plant height **(A)** and dry weight **(B)** of maize under NaCl stress. Different letters indicate significant differences at the *P* < 0.05 level among the different treatments, as determined by LSD *post hoc* comparison tests.

In the pot experiments, we recorded the maize growth index, such as plant height and dry biomass, under salt stress. The results showed that the plant height (*F* = 1.93, *P* < 0.05) and dry weight (*F* = 6.33, *P* < 0.01) were increased significantly by inoculating strains YL07 and YL10 under salt stress conditions. Compared with control plants, the plant height of inoculated strains YL07 and YL10 increased by 12.48 and 21.71%, respectively (Figure 1A), and the dry weight increased by 12.56 and 21.67%, respectively (Figure 1B).

### The Contents of N, K^+^, Na^+^, and K^+^/Na^+^

To determine the absorption of nutrients and main ions in inoculated YL07 and YL10, the contents of N, K^+^, and Na^+^ in maize seedlings were measured. As shown in Table 1, the contents of nutrient N and K^+^ in roots and shoots increased significantly (*P* < 0.05) in inoculated with YL07 and YL10, and the contents of shoots were higher than the contents of roots. Nevertheless, the content of Na^+^ in roots and shoots inoculated with YL07 and YL10 decreased significantly (*P* < 0.05) in comparison with the control treatment. These results showed that the inoculated YL07 and YL10 strains could promote the absorption of N and K^+^ and prevent the absorption of Na^+^. Moreover, the K^+^/Na^+^ ratio is an indicator of ion balance in maize plants. As shown in Table 1, after inoculating the YL07 and YL10 strains, the K^+^/Na^+^ ratio in maize roots and shoots increased significantly (*P* < 0.05) compared with the K^+^/Na^+^ ratio in the control treatment. This result further reveals that inoculated YL07 and YL10 strains could promote the absorption of K^+^ and inhibit the absorption of Na^+^ in maize plants.

**TABLE 1:** Effects of YL07 and YL10 strain inoculation on nutrient elements and essential ion absorption by maize seedlings grown in pots under NaCl stress.

Groups	N content (mg g^–1^ DW)		K^+^ (mg g^–1^ DW)		Na^+^ (mg g^–1^ DW)		K^+^/Na^+^ ratio	
	Shoot	Root	Shoot	Root	Shoot	Root	Shoot	Root
CK3	30.03 ± 2.61b	14.95 ± 0.76b	30.66 ± 3.54b	12.92 ± 0.28c	5.47 ± 0.19a	8.99 ± 0.62a	5.68 ± 0.84b	1.45 ± 0.08b
YL	36.47 ± 1.57a	22.31 ± 1.61a	51.79 ± 1.76a	14.31 ± 0.34b	2.13 ± 0.28b	5.58 ± 0.21b	25.19 ± 3.03a	2.68 ± 0.33a
YL	35.46 ± 0.75ab	23.65 ± 1.76a	56.92 ± 4.43a	16.83 ± 0.56a	2.67 ± 0.28b	5.57 ± 0.32b	21.82 ± 1.56a	3.04 ± 0.17a

*Different letters indicate significant differences at the P < 0.05 level among the different treatments based on one-way ANOVA.*

### Antioxidant Enzymes, GSH, Soluble Sugar, and Proline Content

**FIGURE 2:**
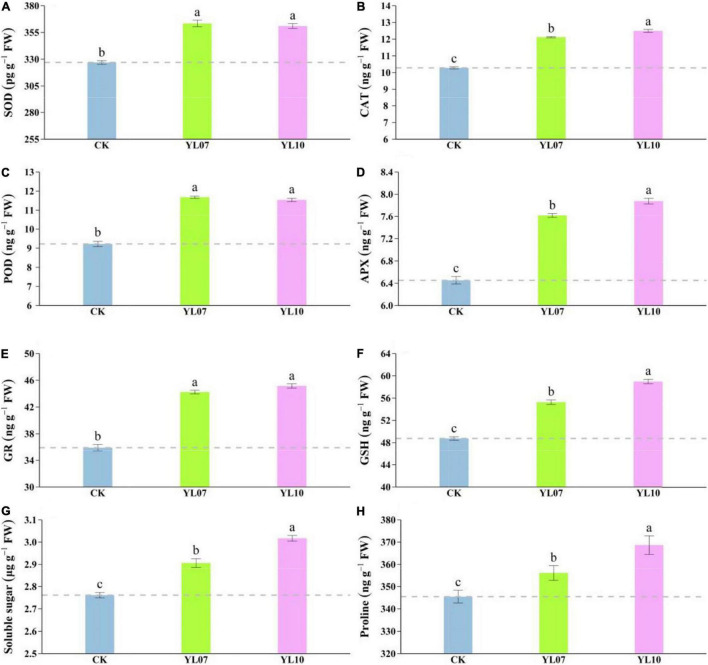
Effect of strains YL07 and YL10 on antioxidant enzymes, including SOD **(A)**, CAT **(B)**, POD **(C)**, APX **(D)**, and GR **(E)** activities of maize under NaCl stress. Effects of YL07 and YL10 inoculation on GSH **(F)**, soluble sugar **(G)**, and proline **(H)** contents of maize grown in pots for 31 days under NaCl stress. Different letters indicate significant differences at the *P* < 0.05 level among the different treatments, as determined by LSD *post hoc* comparison tests. The content of IAA and ABA in plants.

Salt stress mainly triggers the production of reactive oxygen species (ROS), and a large amount of ROS will cause serious damage to plant cells. Antioxidant enzymes such as SOD, CAT, POD, APX, and GR can remove excess ROS to maintain normal plant physiological activities. As shown in Figure 2, the contents of SOD, CAT, POD, APX, and GR in leaves inoculated with the YL07 and YL10 strains dramatically increased compared to the control treatment, in which SOD, YL07 and YL10 increased by 11.13 and 10.42% (*F* = 96.08, *P* < 0.001), respectively; CAT, YL07 and YL10 increased by 17.88 and 21.49% (*F* = 279.60, *P* < 0.001); POD, YL07 and YL10 increased by 26.65 and 25.11% (*F* = 132.10, *P* < 0.001); APX, YL07 and YL10 increased by 18.07 and 22.10% (*F* = 153.10, *P* < 0.001); GR, YL07 and YL10 increased by 23.15 and 25.73% (*F* = 127.60, *P* < 0.001), respectively. Similarly, the GSH content of YL07 and YL10 was significantly enhanced by 13.31 and 20.94%, respectively (*F* = 223.80, *P* < 0.001), compared to the control treatment (Figure 2F). As shown in Figures 2G,H, soluble sugars and proline showed the same results. The inoculated YL07 and YL10 were also considerably increased compared to the control treatment, in which soluble sugar, YL07 and YL10 increased by 5.24 and 9.26%, respectively (*F* = 81.51, *P* < 0.001); proline, YL07 and YL10 increased by 3.07 and 6.71%, respectively (*F* = 11.96, *P* < 0.001).

**FIGURE 3:**
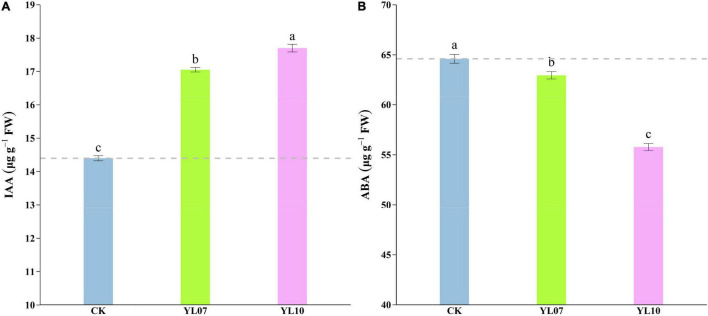
Effect of inoculating with strains YL07 and YL10 on endogenous phytohormone IAA content **(A)** and ABA content **(B)** of maize seedlings grown in pots for 31 days under NaCl stress conditions. Different letters indicate significant differences at the *P* < 0.05 level among the different treatments, as determined by LSD *post hoc* comparison tests.

Under salt stress, the content of plant endogenous hormones regulates a series of physiological and biochemical reactions. As shown in Figure 3, the production of IAA was dramatically enhanced and the level of ABA was reduced in inoculated YL07 and YL10 under NaCl stress. Under inoculation with YL07 and YL10, the IAA content in the leaves of inoculated maize seedlings was significantly (*F* = 447.40, *P* < 0.001) increased by 18.46 and 22.96%, respectively, compared to the IAA content in the leaves of uninoculated control seedlings (Figure 3A). In contrast, the ABA content in maize leaves was significantly (*F* = 158.30, *P* < 0.001) decreased by 2.55 and 13.67%, respectively, under inoculation with the YL07 and YL10 treatments compared to the control treatments (Figure 3B).

### Expression of Related Genes in Maize Seedlings

**FIGURE 4:**
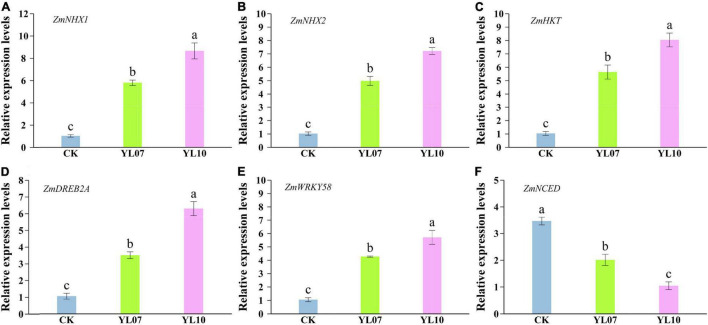
Effect of inoculating with strains YL07 and YL10 on the expression of genes including ZmNHX1 **(A)**, ZmNHX2 **(B)**, ZmHKT **(C)**, ZmDREB2A **(D)**, ZmWRKY58 **(E)**, and ZmNCED **(F)** related to salt tolerance in maize seedlings under NaCl stress conditions. Different letters indicate significant differences at the *P* < 0.05 level among the different treatments, as determined by LSD post hoc comparison tests.

As shown in Figures 4A–C, the transcription levels of the ion balance-related genes *ZmNHX1* and *ZmNHX2* and *ZmHKT* in inoculated YL07 and YL10 under NaCl stress were upregulated in comparison with the control treatments, in which *ZmNHX1*, YL07 and YL10 increased by 465.04 and 742.80% (*F* = 76.90, *P* < 0.001), respectively; *ZmNHX2*, YL07, and YL10 increased by 385.20 and 602.87% (*F* = 150.60, *P* < 0.001), respectively; and *ZmHKT*, YL07, and YL10 increased by 442.57 and 673.70% (*F* = 68.13, *P* < 0.001), respectively. Similarly, key transcription gene *ZmDREB2A* and *ZmWRKY58* levels linked to plant response to abiotic stress were also upregulated inoculated with YL07 and YL10 in which *ZmDREB2A*, YL07 and YL10 increased by 232.13 and 497.66% (*F* = 82.28, *P* < 0.001), respectively (Figure 4D); *ZmWRKY58*, YL07 and YL10 increased by 305.60 and 441.68% (*F* = 57.79, *P* < 0.001), respectively (Figure 4E). Moreover, the expression level of the key gene *ZmNCED*, which is associated with the ABA biosynthesis pathway, indicated that the YL07 and YL10 strains dramatically reduced the ABA content (Figure 4F). Analogously, the expression levels of *ZmNCED* were significantly decreased by 41.96 and 69.77% (*F* = 51.92, *P* < 0.001) under inoculated YL07 and YL10, respectively, compared to the control treatments.

### Correlation Analysis Between the Studied Parameters

**FIGURE 5:**
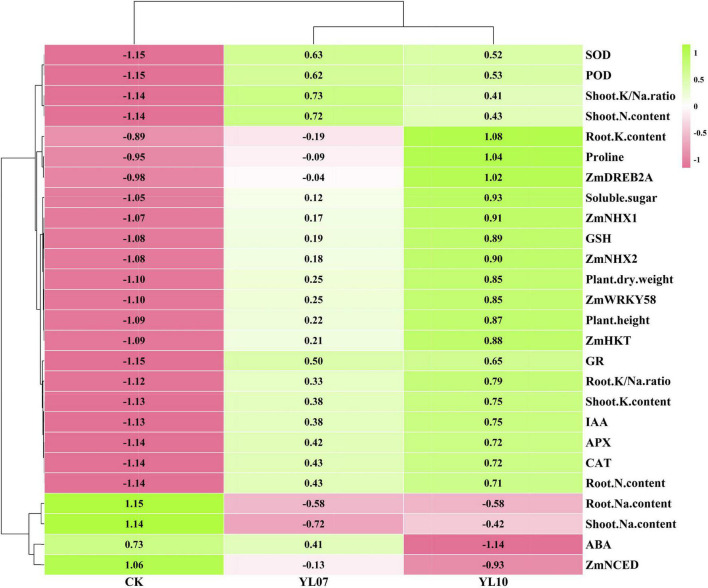
Hierarchical clustering to understand treatment-variable relationships in maize plants under NaCl stress conditions.

**FIGURE 6:**
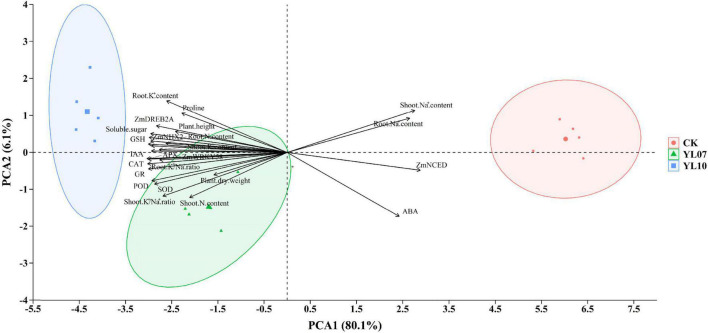
Principal component analysis of all indicators in maize plants under salt stress.

Cluster analysis of all studied parameters revealed that all samples clustered into two major clusters (Figure 5). The changes in the parameters were further observed in the heatmap and indicated that the different treatment groups could be separated based on these parameters. The PCA of all parameters revealed the effects of uninoculated CK and inoculation YL07 and YL10 on maize plants under salt stress (Figure 6). PC1 accounted for 80.1% of the variance, and PC2 accounted for 6.1% of the variance. The CK treatment group was differentiated from the YL07 and YL10 groups, which might be attributed mainly to changes in parameters, including Na^+^ content, ABA level, and *ZmNCED* expression level.

## Discussion

In previous studies, we found that fungi were more vulnerable to salt than bacteria in Xinjiang saline–alkali land (Hou et al., 2021). Therefore, in this screening of plant growth-promoting rhizobacteria, we focused on bacteria. Second, the previous screening methods were first based on the secretion characteristics of bacteria (Arruda et al., 2013; Pandey and Gupta, 2020; Sultana et al., 2021), but we first started from the germination rate experiment of maize by bacteria. On the one hand, our procedure was more instructive from the perspective of production practice. On the other hand, we found that bacteria secreting promoting substances (such as indole acetic acid, solubilized phosphates, siderophores, and extracellular polysaccharides) might not necessarily promote the growth of crops (Arruda et al., 2013; Wang W. F. et al., 2018). Based on these reasons, combined with the results of the germination experiment and salt tolerance results by bacteria, we comprehensively selected the two kinds of dominant strains, precisely measured the growth-promoting substance secreted by bacteria, and finally thoroughly explained the growth-promoting function of bacteria on maize at the mechanistic level.

### Characterization of Plant Growth-Promoting Rhizobacteria Traits Under Salt Stress

In this study, we isolated culturable rhizosphere bacteria from maize rhizosphere soil in saline–alkali land of Xinjiang for the first time and studied their ability *in vitro*. Two PGPR strains, *Bacillus atrophaeus* WZYH01 (YL07) and *Planococcus soli* WZYH02 (YL10), were capable of producing IAA and EPS and solubilizing phosphate effectively (Supplementary Figure 3). *Bacillus atrophaeus* belongs to the *Bacillus* genus, which is a gram-positive, aerobic, spore-forming bacterium phenotypically (Nakamura, 1989). *Bacillus atrophaeus* includes many strains that play an important role in the production of a range of products, including fermented food, bioinsecticides, antibiotics, and other products with commercial applications (Schallmey et al., 2004). A study showed that *B. atrophaeus* was remarkably resistant to extreme environments (i.e., heat, radiation, toxic chemicals, and pH extremes) (Moir, 2006). Ma et al. (2018) reported that *B. atrophaeus* strain GQJK17, as a kind of PGPR, had significant effects on inhibiting some soil-borne diseases and promoting the growth of some plants. *Bacillus atrophaeus* was verified to promote the growth of *Zea mays* L. and *Solanum lycopersicum* and had extraordinary activity in root colonization (Huang et al., 2015). The genus *Planococcus* was first proposed by Migula to adapt to accommodate gram-positive, aerobic bacteria of the family Planococcaceae (Yang et al., 2020). Many *Planococcus* species have been isolated from a variety of natural environments, including soil samples, marine samples, and extreme environments (Yang et al., 2020). They were highly resistant to the environment, usually salt-tolerant, and could grow at lower temperatures; some special strains could even grow at −15°C (Zhang et al., 2021). For example, *Planococcus* has also shown the ability to degrade and treat various pollutants, such as phenols and heavy metals (Sun et al., 2014). *Planococcus rifietoensis* M8^T^ is a salt-tolerant bacterium with potential plant growth-promoting properties that can promote the growth of the wheat by converting ammonia into nitrogen (See-Too et al., 2016). Although the above literature evidence indicates that some species belonging to the *Bacillus* and *Planococcus* genera have appropriate potential as PGPR, to our knowledge, this is the first report that *B. atrophaeus* and *Planococcus soli* can produce IAA, EPS, and solubilized phosphate under salt stress, which can be used as PGPR to promote maize plant growth under salt stress.

Plant growth-promoting rhizobacteria can effectively improve the tolerance of plants to abiotic stress and then promote plant growth and yield. In our results, we observed that maize inoculated with *B. atrophaeus* WZYH01 and *Planococcus soli* WZYH02 showed better growth prospects under salt stress. For example, in the germination experiment, the strains *B. atrophaeus* WZYH01 and *Planococcus soli* WZYH02 reduced the adverse effect of salt stress on the maize germination rate compared with the control (Supplementary Figure 1A). Inoculation with *B. atrophaeus* WZYH01 and *Planococcus soli* WZYH02 also increased maize root length compared with the root length in the uninoculated treatment (Supplementary Figure 1B). Our research was consistent with a previous study showing that the application of PGPR strains significantly improved the percentage of seed germination under saline conditions (Kaymak et al., 2009).

In the pot experiment, we also found that the strains *B. atrophaeus* WZYH01 and *Planococcus soli* WZYH02 had a certain alleviating effect on the growth of maize under salt stress. Compared with the uninoculated control, inoculating strains markedly increased the maize plant height and dry weight (Figure 1). Consistently, Mayak et al. (2004) indicated that salt-tolerant PGPR bacteria could improve the salt tolerance and yield of tomato, corn, and pepper crops in saline soil land.

Plant growth-promoting rhizobacteria traits, such as hormone production, phosphate solubilization, EPS secretion, ACC deaminase synthesis, siderophore production, and nitrogen fixation, had positive effects on promoting plant growth (Etesami and Maheshwari, 2018). This study clearly stated that both *B. atrophaeus* WZYH01 and *Planococcus soli* WZYH02, which produce hormone IAA, solubilize phosphorus, and secrete EPS, could improve the maize germination rate, root length, and growth conditions. Therefore, with the inoculation of these two strains, the promoting effect of maize under salt stress might be mediated by these growth-promoting characteristics. The hormone IAA secreted by bacteria was suggested to play an important role in promoting the growth of maize. Kumari et al. (2015) indicated that the higher root length of the inoculated plants might be due to the existence of the IAA hormone secreted by bacteria. Several studies also reported that PGPR strains showed IAA activity, and the interaction between IAA and plants helped to increase root growth under salt stress, which might be an adaptive response to salt stress (Albacete et al., 2008).

Bacterial polysaccharides synthesized and secreted into the external environment may be referred to as exopolysaccharides (Nwodo et al., 2012). EPS produced by PGPR contributed to soil aggregation, water retention, and chelation of metal ions in plants under salinity (Upadhyay et al., 2011). Under salt stress, EPS produced by PGPR could combine with Na^+^ ions, reduce Na^+^ content, and help to maintain ion balance in the root zone (Mishra et al., 2021). Liu et al. (2017) found that EPS produced by *B. amyloliquefaciens* FZB42 could finally reduce the Na^+^ concentration in plants owing to binding to Na^+^ content and inhibiting the absorption of Na^+^. In the current study, *B. atrophaeus* WZYH01 and *Planococcus soli* WZYH02 produced a large number of EPS (Supplementary Figure 3), which could help maize plants resist salt stress. A previous study indicated that the formation of EPS and biofilms produced by the PGPR *Pseudomonas anguilliseptica* SAW 24 significantly improved the growth of *Vicia faba* L. under salt stress (Mohammed, 2018).

Phosphorus is the second most important key plant nutrient after nitrogen (Nanthakumar and Panneerselvam, 2017). Phosphorus participates in the important functions of plant energy metabolism, structural function, and signal transduction function and is necessary for plant growth and development (Lobo et al., 2019). Most of the phosphorus in the soil was fixed. Therefore, although the soil was rich in inorganic phosphorus and organic phosphorus, plant-available phosphorus was rarely effective. Phosphate solubilizing bacteria (PSB) have the ability to mobilize insoluble phosphate in soil and improve the tolerance of plants to various stresses (Kumar et al., 2018). The available phosphorus is dissolved by phosphate-dissolving microorganisms by producing acids (Alori et al., 2017). In this investigation, *B. atrophaeus* WZYH01 and *Planococcus soli* WZYH02 were solubilized phosphorus strains, which could help maize overcome nutrient deficiency and hormone imbalance under salt conditions (Supplementary Figure 3). Consistently, PSB could increase the content of soluble phosphorus in soil solution, promote the root growth of rice seedlings, and increase plant biomass (Panhwar et al., 2013). In addition, cucumber plants inoculated with *Cucumis sativus* also exhibited a higher P content than uninoculated plants under salinity stress (Kang et al., 2014a).

### Effect of Plant Growth-Promoting Rhizobacteria Inoculation on Maize Tolerance to NaCl Stress

Salt stress inhibited plant growth due to the increase in Na^+^ concentration and the decrease in the K^+^/Na^+^ ratio. Na^+^ exclusion and K^+^ influx are the most important strategies for plants to alleviate salt stress (Rojas-Tapias et al., 2012). PGPR could increase intracellular K^+^ levels and maintain the K^+^/Na^+^ ratio by removing excess Na^+^ in plant cells under salt stress (Mishra et al., 2021). We observed that high levels of Na^+^ in maize roots and shoots were negatively correlated with maize biomass and plant height. In contrast, the K^+^ content was positively correlated with plant biomass and plant height (Figure 6). Similarly, the results showed that inoculation with the two bacteria resulted in a decrease in Na^+^ and a significant increase in K^+^, which led to an increase in the K^+^/Na^+^ ratio (Table 1). Consistently, under salt stress, inoculation of PGPR has been reported to be able to avoid excessive accumulation of Na^+^ in plants and maintain ion homeostasis (Kang et al., 2014b). Moreover, PGPR *Azospirillum lipoferum* and *Azotobacter chroococcum* increased the K^+^ level of maize plants under salt stress, maintained the K^+^/Na^+^ ratio, and reduced the Na^+^ level (Latef et al., 2020).

The main aspects of PGPR-mediated plant salt tolerance include generating reaction mechanisms to concentrate toxicity and establish osmotic equilibrium to avoid plant cell desiccation and flaccidity. As part of this regulatory pathway, Na^+^/H^+^ antiporters of tonoplast are encoded by the *NHX* genes. This progress in understanding the signal transduction pathway for the ion homeostasis and salt resistance of higher plants opens possibilities to establish salt-resistant crop plants (Zorb et al., 2005). PGPR inhibits the absorption of Na^+^ by changing the composition of the cell wall or cell membrane, increases the electrogenic Na^+^/H^+^ ion transporters in plants, and increases the expression of *NHX* transporters (Kumar Arora et al., 2020). In this study, inoculating two strains, *B. atrophaeus* WZYH01 and *Planococcus soli* WZYH02, significantly reduced the level of Na^+^ and upregulated the expression of *ZmNHX* and *ZmHKT* genes in maize under salt stress, indicating that inoculated strains might reduce the content of Na^+^ in maize by isolating Na^+^ into vacuoles and excreting Na^+^ from cells to improve Na^+^ toxicity and salt tolerance (Figures 4A–C). A previous study indicated that *Bacillus* promoted the growth of maize under salt stress by upregulating the expression of the plant ion homeostasis-related genes *NHX1* and *HKT1*. Furthermore, *Bacillus subtilis* reduced Na^+^ uptake by *Arabidopsis thaliana* roots by downregulating the high-affinity K^+^ transporter (*HKT1*) under salt stress (Zhang et al., 2008). Our results substantiate that the two PGPRs could improve maize salt tolerance by the upregulation of *ZmNHX* and *ZmHKT* genes with increased inclusion of Na^+^ in vacuoles to avoid the accumulation of Na^+^.

### Mechanism of Plant Growth-Promoting Rhizobacteria in Promoting Maize Growth

The plant responds to salt stress conditions by generating reactive oxygen species (ROS), which generate oxidative stain in plants and impair chlorophyll, DNA, protein, and membrane functions (Kaushal and Wani, 2016). Salinity also causes the deleterious effect of osmotic pressure, deterioration of metabolic functions, decreased energy requirements, and disruption of cell divisions as well (Desoky et al., 2019). Whenever possible, plants are developing and/or adopting an antioxidant system to mitigate the destructive effects caused by salinity-induced ROS (Desoky et al., 2020). This system contains many enzymatic and non-enzymatic antioxidants with low molecular weights (Mishra et al., 2021). In the current study, inoculation with *B. atrophaeus* WZYH01 and *Planococcus soli* WZYH02 significantly increased the activity of enzymatic antioxidants (SOD, CAT, POD, APX, and GR) and the content of the non-enzymatic antioxidant GSH in maize under salt stress (Figures 2A–F). The above results showed that the two strains could stimulate the defense response of plants to reactive oxygen species detoxification and promote the growth of maize seedlings. Enzymatic and non-enzymatic antioxidant activities may be reduced as PGPR detoxifies ROS through a variety of plant protection mechanisms, such as K^+^ accumulation and phytohormones secreted by PGPR.

Plant growth-promoting rhizobacteria treatment resulted in a significant increase in proline and soluble sugars contents in salt stress, which acted as an osmoprotectant mechanism (Figures 2G,H). Proline, as a non-enzymatic antioxidant, increases with PGPR inoculation to improve the plant antioxidant system and restore energy compensation in plants. Proline reduces ROS damage and enhances plant tolerance by reducing the detoxification of ROS resulting from salinity stress (Howladar, 2014). Likewise, the accumulation of soluble sugars maintains the harmony between the osmotic quality of the cytosol and the vacuole. However, Desoky et al. (2020) indicated that proline and soluble sugars were significantly reduced by inoculation with PGPRs, which was not consistent with our research. In the present study, *B. atrophaeus* WZYH01 and *Planococcus soli* WZYH02 could increase proline and soluble sugar accumulation under salt stress. This also suggested the existence of other osmoprotectants to improve plant resistance to salt stress.

Plant growth-promoting rhizobacteria have been reported to stimulate the plant’s nutrient acquisition machinery by activating nutrient-deficiency-induced transcription factors in addition to auxins and cytokinin secretion (Li et al., 2020). ABA is involved in various physiological processes of plants, including stomatal conductance, seedling growth, and plant responses to environmental stress (Li et al., 2020). Mishra et al. (2021) indicated that inoculation with PGPR could change the expression of ABA and the related gene *NCED* in plants. PGPR *P. fluorescence* and *P. putida* could downregulate the NCED gene of ABA biosynthesis in salt-stressed barley (Zaib et al., 2020). This study was consistent with our research. *Bacillus atrophaeus* WZYH01 and *Planococcus soli* WZYH02 showed a lower ABA content and *ZmNCED* expression (Figures 3B, 4F). *DREBs* and *WRKY* transcript factors also play a critical role in enhancing plant salt tolerance (Li et al., 2020). Similar to the situation in wheat, the expression of a *DREB2* homolog in maize was found to be activated also by cold despite its induction by drought and salt stress (Nguyen et al., 2009). This study showed an increase in *ZmDREB2A* and *ZmWRKY58* gene expression in *B. atrophaeus* WZYH01 and *Planococcus soli* WZYH02-inoculated maize under saline conditions compared to non-inoculated control plants (Figures 4D,E). Therefore, under salt stress, *B. atrophaeus* WZYH01 and *Planococcus soli* WZYH02 are involved in changes in plant hormone-related gene expression, which may be involved in the improvement of salt stress and the regulation of plant development.

## Conclusion

In this study, two salt-tolerant strains, *B. atrophaeus* WZYH01 and *Planococcus soli* WZYH02, were isolated from maize rhizosphere soil in Xinjiang, China. Two strains could tolerate up to 1197 mM NaCl and exhibited three growth-promoting traits: IAA, exopolysaccharide production, and phosphate solubilization. *Bacillus atrophaeus* WZYH01 and *Planococcus soli* WZYH02 could protect maize from salt stress, which could be considered the integration of multiple physiological processes, including improving plant mineral nutrition and increasing plant antioxidant capacity through plant hormones (IAA and ABA), and the expression of transporter genes, increasing the K^+^/Na^+^ ratio in maize plants. In general, the effect of *Planococcus soli* WZYH02 was more prominent in the growth index and gene expression than the effect of *B. atrophaeus* WZYH01. In conclusion, this study showed that *B. atrophaeus* WZYH01 and *Planococcus soli* WZYH02 could be used as eco-friendly PGPR based on their potential in promoting and protecting maize plant growth under saline conditions.

## Data Availability Statement

The datasets presented in this study can be found in online repositories. The names of the repository/repositories and accession number(s) can be found below: National Center for Biotechnology Information (NCBI) BioProject database under accession numbers MZ919348 and MZ919345.

## Author Contributions

YH involved in investigation, data curation, writing—original draft preparation, and visualization. WZ involved in conceptualization, methodology, writing—review and editing, funding acquisition, and supervision. CA took a leading role in writing—review and editing. YL involved in investigation, validation, and formal analysis. ZW and MH designed the methodology and formal analysis. JH involved in funding acquisition and supervision. All authors contributed to the article and approved the submitted version.

## Conflict of Interest

The authors declare that the research was conducted in the absence of any commercial or financial relationships that could be construed as a potential conflict of interest.

## Publisher’s Note

All claims expressed in this article are solely those of the authors and do not necessarily represent those of their affiliated organizations, or those of the publisher, the editors and the reviewers. Any product that may be evaluated in this article, or claim that may be made by its manufacturer, is not guaranteed or endorsed by the publisher.

## Footnotes

1
http://english.moa.gov.cn

2
http://www.kcscin.com
