# Supplementary material for: Bacillus atrophaeus WZYH01 and Planococcus soli WZYH02 Improve Salt Tolerance of Maize (Zea mays L.) in Saline Soil
Source: Front Plant Sci. 2022 May 6;13:891372. doi: 10.3389/fpls.2022.891372 (PMC9121094; doi:10.3389/fpls.2022.891372)
Supplement: Supplementary file 1 [file Data_Sheet_1.docx]

**Supplementary Materials**

[1. Screening methods 1](#_Toc100093466)

[1.1 Screening of strains from maize rhizosphere soil 1](#_Toc100093467)

[1.2 Preparation of strain inoculum and pretreatment of maize seeds 1](#_Toc100093468)

[1.3 Germination rate test 1](#_Toc100093469)

[1.4 Strain salt tolerance test 2](#_Toc100093470)

[1.5 Characterization of the isolates 2](#_Toc100093471)

[2. Results 1](#_Toc100093472)

[2.1 Germination test 1](#_Toc100093473)

[2.2 Bacterial tolerance to NaCl 2](#_Toc100093474)

[2.3 Identification and growth-promoting characteristics of the isolate 2](#_Toc100093475)

[3. Supplementary tables 5](#_Toc100093476)

[Table S1 Characteristics of the primers used in the qRT-PCR for studying the relative expression of genes in maize lines under salt stress. 5](#_Toc100093477)

# Screening methods

## 1.1 Screening of strains from maize rhizosphere soil

Samples of maize rhizosphere soil (soil adhered to roots) were collected from Yanqi, Xinjiang Province, China (41°91′ N, 86°49′ E, elevation 1061 m). At the location, root samples from 5 plants were obtained by digging out part of a clump to a depth of approximately 20 cm. Soil from the rhizosphere of five plants was mixed to obtain one pooled sample per location for chemical analysis. The soil physicochemical properties: saturated electrical conductivity, 6.13 ds/m; pH, 8.32; clay content, 0.9%; silt content, 32.47%; sand content, 66.63%; soil organic matter, 5.49 g/kg; available nitrogen, 16.34 mg/kg; available phosphorus, 25.33 mg/kg; and available potassium, 109.67 mg/kg.

Collected maize plants with intact roots and adhering soil were transported to the laboratory in sterile bags where the rhizosphere soil was separated from the roots and processed for the isolation of rhizosphere growth-promoting bacteria. Five grams of rhizosphere soil was suspended in 50 ml sterile phosphate buffer solution (c=1/15 M, pH = 7.2) and incubated at 28 °C with shaking for 24 h. Aliquots (0.1 ml) from serially diluted samples (up to 10^-6^) were added to LB solid medium and then placed in a constant temperature incubator for 16 h at 28 °C. According to color, shape and size characteristics on the Petri dish, single bacterial colonies with different characteristics were selected. Three-paragraph lines were scribed on LB solid medium and then cultured in a constant temperature incubator at 28 °C to obtain pure colonies. Subsequently, monoclonal purified bacteria were cultured in 855 mM NaCl LB (Luria-Bertani) solid medium to capture salt-tolerant bacteria. Eventually, thirteen pure-culture colonies were preserved in 80% glycerol and stored at −80 °C. All the above experiments were carried out in a sterile environment.

## 1.2 Preparation of strain inoculum and pretreatment of maize seeds

The strains were cultured in a 100 ml Erlenmeyer flask with 30 ml LB liquid medium at 28 °C with shaking at 220 rpm for 16 h and then centrifuged at 7,000 rpm for 5 min. The supernatant was discarded, the strain at the centrifuge tube bottom was resuspended in phosphate buffer solution (c=1/15 M, pH = 7.2), and the absorbance of the suspension was adjusted to OD_600_ = 0.8. Maize seeds were treated with 75% ethanol for 30 s, sterilized with 10% sodium hypochlorite solution for 15 min, and then washed with sterile water 5-6 times. Sterilized seeds were soaked in sterile water for 12 h, then dipped in strain suspension (OD_600_ = 0.8) for 2 h and for the control group, only dipped in phosphate buffer solution for 2 h. All the above experiments were carried out in a sterile environment. Seeds inoculated with thirteen strains were used for subsequent operations.

## 1.3 Germination rate test

Fifty sterilized seeds under each inoculated seed (operated according to “Materials and Methods 2.2”) were picked and placed in a 12 cm sterile Petri dish with 15 ml 171 mM NaCl solution. The Petri dish was placed in an artificial climate incubator (Wuhan Ruihua Instrument and Equipment Co., Ltd., Hubei Province, China). The germination rate test conditions were as follows: temperature, 25 °C; humidity, 60%; light duration, 12 h/12 h day/night. The germination number was recorded on the 7^th^ day. Each strain treatment had 7 replicates, totaling 98 Petri dishes.

Root length was measured on the 7^th^ day of the germination test (i.e. 180 h after sowing). According to axial root length, the roots can be divided into three groups: < 4 mm, short root; 4-8 mm, middle root; > 8 mm, long root. Then, 3 seeds were randomly selected from each group to measure the root length; that is, 9 maize root lengths were measured in each Petri dish (i.e. 63 maize roots were measured in each treatment).

## 1.4 Strain salt tolerance test

The optical density at 600 nm of 13 strains was adjusted to 0.8, and 100 µl (10% v/v) of bacterial solution was inoculated into test tubes containing 10 mL LB liquid medium supplemented with 171, 342, 513, 684, 855, 1026 and 1197 mM NaCl. Then, the tubes were placed on a shaker at 28 °C and 220 rpm for 24 h. Subsequently, the optical density at 600 nm was measured. Each strain treatment at each NaCl concentration had 7 replicates, totaling 637 test tubes. Strain growth-promoting traits including indole acetic acid (IAA), exopolysaccharides (EPS) and phosphate solubilization (PS) were measured, which use *E.coli* as control treatment.

## 1.5 Characterization of the isolates

For 16S rRNA (partial 16S ribosomal RNA) gene sequence analysis, isolated strains were streaked on the LB plates and the resulting bacterial colonies were used as templates to amply 16S rDNA. PCR program used the following universal primers: 1492R: 5’-CAC GGA TCC TAC GGG TAC CTT GTT ACG ACT T T-3’ and 27 F: 5’-GTG CTG CAG AGA GTT TGA TCC TGG CTC AG-3’. The amplification protocol was under the following conditions: the PCR program included an initial denaturation at 94 °C for 5min; 30 cycles of 94 °C for 30 s, 55 °C for 45 s, and 72 °C for 90 s; and the final extension of 72 °C for 10 min. The amplified PCR products were revaluated by gel electrophoresis using 1% agarose gel, then visualized under short-wavelength UV light stained. The PCR products were sent to Tsingke Biotechnology Co., Ltd. [(https://www.tsingke.com.cn)](https://www.tsingke.com.cn/) for nucleotide sequencing by the Sanger dideoxy sequencing method. Sequences were aligned to their nearest neighbors with Mega X software (version 10.0.5), and then by neighbor-joining (NJ) algorithms. The phylogenetic tree was realized by using the program MEGA-X.

# Results

## 2.1 Germination test

Under 171mM NaCl stress conditions, the germination rates of maize seeds did differ significantly among treatments (F = 36.81, *P <* 0.001). On the 7^th^ day, the germination rates of YL01, YL05, YL06, YL07, YL09, YL10, and YL13 increased by 135.59%, 120.34%, 106.78%, 127.12%, 103.39%, 172.89% and 111.86%, respectively (**Fig. S1A**). In contrast, the germination rates of YL02, YL03, YL04, YL11 and YL12 maize seeds were lower than the germination rates of the uninoculated group.

As shown in **Fig. S1B**, inoculation with bacteria significantly influenced maize root length (F = 37.78, *P <* 0.001), of which YL01, YL05, YL06, YL07, YL08, YL09, YL10 and YL13 significantly increased the root length of maize by 145.34%, 93.29%, 123.23%, 171.28%, 13.46%, 191.18%, 73.95% and 98.48%, respectively. However, YL02, YL03, YL04, YL11 and YL12 had negative effects on maize root length and significantly reduced the root length of the maize plants.

**

**

**
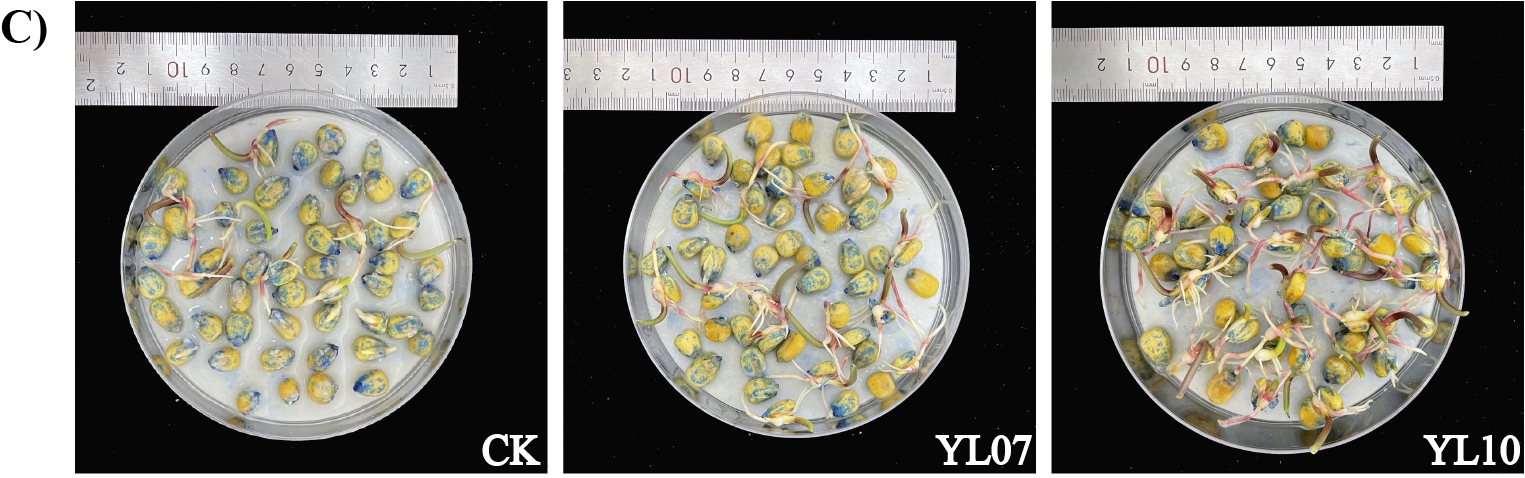
**

**Fig. S1.** Germination rates of maize seeds inoculated with strains YL01-YL13 on the seventh day **A)** and root lengths **B)** under NaCl stress. Germination pictures **C)** were taken on the 7th day. Bars represent the means ± SE of seven replicates. Different letters indicate significant differences at the *P* < 0.05 level among the different treatments, as determined by LSD post hoc comparison tests.

## 2.2 Bacterial tolerance to NaCl

As shown in **Fig. S2A** and **Fig. S2B**, strains YL01-YL13 exhibited different tolerances to salt. As the salt content increased, the reproductive capacity of YL07, YL09, YL10 and YL13 increased to a certain degree. Nevertheless, the reproductive capacity of YL01, YL02, YL03, YL04, YL05, YL06, YL08, YL11 and YL12 decreased gradually with the increase in salt content, of which the optical densities at 600 nm of YL01, YL03 and YL12 were all less than 1 under the whole salt treatment and YL06 decreased sharply with 342 mM salt concentration. Therefore, combined with the germination rate test and salt tolerance test results, we selected YL05, YL07, YL08, YL09, YL10 and YL13 as the target strains for subsequent

**


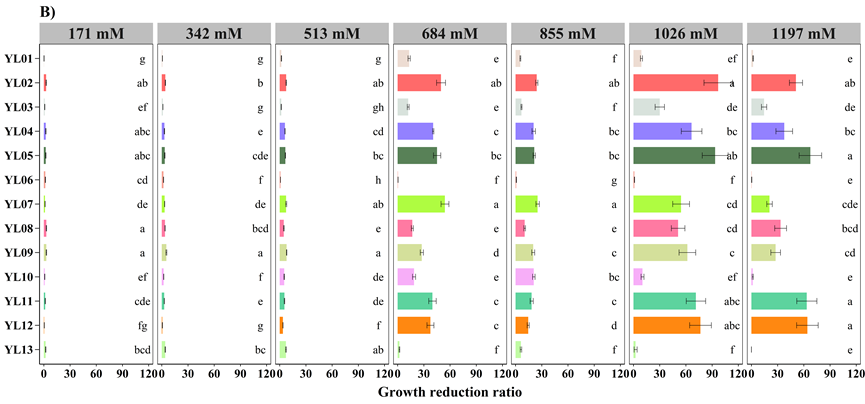
**

**Fig. S2.** Effect of different NaCl concentrations on YL01-YL13 bacterial growth **A)**; Growth reduction ration of different strains under salt stress compared *E-coli* **B)**. Bars represent the means ± SE of seven replicates. Different letters indicate significant differences at the *P* < 0.05 level among the different treatments, as determined by LSD post hoc comparison tests.

## 2.3 Identification and growth-promoting characteristics of the isolate

The strains *E.coli*, YL05, YL07, YL08, YL09, YL10 and YL13 were examined for their plant growth-promoting traits. As shown in **Fig. S3**, the indices including IAA, EPS and PS, were significantly different among the 6 strains. IAA concentration: YL07 > YL10 > YL13 > YL08 > YL05 > YL09 > *E.coli* (**Fig. S3A**); EPS content: YL10 > YL07 > YL08 YL13 > YL05 > YL09 > *E.coli* (**Fig. S3B**); PS production: YL07 > YL13 > YL10 > YL08 > YL05 > YL09 > *E.coli* (**Fig. S3C**). In summary, YL07 and YL10 were selected as the research objects of rhizosphere growth-promoting bacteria.

**

**

**Fig. S3.** Plant growth-promoting properties of six rhizosphere bacteria for the IAA content **A)**, EPS content **B)** and phosphate solubilization (PS) production **C)**. Different letters indicate significant differences at the *P* < 0.05 level among the different treatments, as determined by LSD post hoc comparison tests.

According to the results of 16S rRNA sequencing, the sequences of the YL07 and YL10 strains were compared with available 16S rRNA sequences of related species from the NCBI databank. The sequences were submitted to GenBank with the accession numbers MZ919348 and MZ919345. The phylogenetic tree constructed by MEGA-X software showed that YL07 named *Planococcus soli* WZYH02 and *Planococcus soli* NJC23, *Planococcus soli* c23 C1146 shared high homology (**Fig. S4A**); YL10 named *Bacillus atrophaeus* WZYH01 and *Bacillus atrophaeus* B4144 201601 NODE 12 shared high homology (**Fig. S4B**).


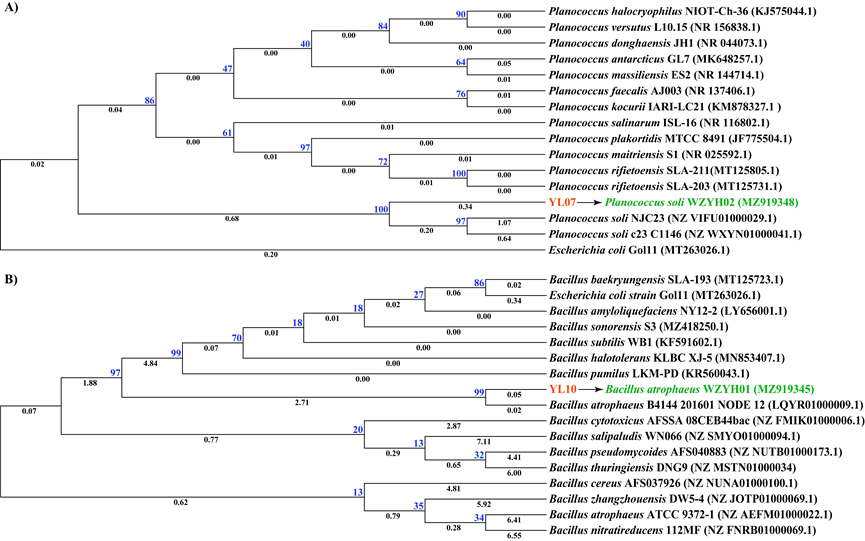


**Fig. S4.** Phylogenetic tree of the strains YL07 and YL10 based on 16S rRNA gene sequences.

# Supplementary tables

| Table S1 Characteristics of the primers used in the qRT-PCR for studying the relative expression of genes in maize lines under salt stress. | | |
| --- | --- | --- |
| Description | Forward primer (5’-3’) | Reverse primer (5’-3’) |
| *ZmNHX1* | TAGGTGGGTGAACGAGTCCACC | GTCTCCCAGTTCAAGTGCGCCG |
| *ZmNHX2* | CGAGCCACATACTTGTGTTCGATG | GTTTCATCCTGGTTAAGCACCTG |
| *ZmHKT* | CAGCTCCTCCTCATGACCCTCCTT | AATCGCCATGGCCGTCCTTGTTG |
| *ZmDREB2A* | GGCTGAGCGCAACAAGCATTTGG | CCGGGGAAGTTAGTCCGTGCC |
| *ZmWRKY58* | GCAGAAGACAGAATCCAGGGTTC | GTTGCTTCTCTTGAACATCTC |
| *ZmNCED* | GCGCAACGGCGCCAACCCCTGC | GTTGTAGACGAGGCCGGCGTTG |
| *Actin* | CAGTGGTCGAACAACGGGTATG | CCTGTTCATAATCAAGGGCAACG |
